# The link between atopic dermatitis and asthma- immunological imbalance and beyond

**DOI:** 10.1186/s40733-021-00082-0

**Published:** 2021-12-15

**Authors:** Martina Yaneva, Razvigor Darlenski

**Affiliations:** 1 Department of Dermatology and Venereology, Acibadem City Clinic, Sofia, Bulgaria; 2 Section of Dermatovenereology, Trakia University, Stara Zagora, Bulgaria

**Keywords:** Atopy, Atopic march, Comorbidities, Asthma, Endotypes, Phenotypes

## Abstract

Atopic diseases are multifactorial chronic disturbances which may evolve one into another and have overlapping pathogenetic mechanisms. Atopic dermatitis is in most cases the first step towards the development of the atopic march and represents a major socio-economic burden in the industrialized countries. The treatment of atopic diseases is often long-lasting and in some cases with lower effectiveness than expected.

In order to prevent the development of the atopic march, the links between the atopic diseases have to be understood. The aim of this review is to present some major points outlining the link between atopic dermatitis and asthma, through a research in the medical literature from recent years.

Stratifying patient populations according to the clinical phenotype of their disease and according to specific measurable values (biomarkers) can help to establish the main etiopathogenetic mechanisms of the disease in these populations. This will add predictive value for the evolution of the disease, and will allow the use and research of more targeted therapy in order to stop this evolution and comorbidities.

## Background

Atopic dermatitis (AD), or atopic eczema, is the most common chronic inflammatory disorder [1] which according to the World Health Organization (WHO) affects more than 230 million people worldwide, and is the fourth leading cause of non-fatal disability [2]. AD currently affects between 15 and 20% of the children and between 1 and 10% of the adult population. [3].

Asthma is a long-term condition affecting both children and adults. Around 300 million people worldwide have asthma and it is estimated that by 2025 another 100 million will be affected [4]. Atopic asthma is the most common form of asthma, from which 70–90% of the children and 50% of the adult patients are affected. [5].

Disrupted skin barrier, genetics, allergic sensitization, elevated IgE levels, microbiome, Th2 immunity, environmental triggers are familiar concepts all of which are somehow connected, and their connection outline the concept of atopy and the development of the atopic march.

The aim of this review is to define the main links between atopic dermatitis (AD) and asthma. Through an investigation of the medical literature we will try to present the current understanding of the atopic march and its comorbidities beyond the immunological imbalance concept.

## Search methodology

A systematic literature review was conducted using the online PubMed MEDLINE database. The keywords included “asthma”, “atopic dermatitis”, “atopy”, “skin microbiome”, “atopic march”, “comorbidities”,” skin barrier”. Studies and reviews published in English were included. Non-English articles, animal studies and in vitro experiments were excluded.

The oldest article reviewed was published in 1989 and the newest in 2021.

## Atopic march

AD is associated with food allergy (FA), asthma, and allergic rhinitis (AR), with or without the occurrence of elevated IgE- levels [6]. This gradual transition of one atopic disease into another in an almost specific age range is known as atopic march. AD is considered the first step as disrupted skin barrier, inflammation and bacterial dysbiosis [7] lead to sensitization required for the development of other atopic diseases [8]. However, AD can follow asthma and AR. FA develops early in life, it can precede or follow the AD, and may in some cases be the first sign of the atopic march. FA often occurs before the appearance of AR or asthma [9, 10]. Dysfunctional skin barrier in AD could lead to food sensitization or FA could accompany AD. Either way food sensitization could be a marker for AD severity, an endotype, which could suggest more probable transition to other atopic comorbidities (asthma and AR) [11]. In the classical evolution of the atopic march allergic asthma is the result of hyperreactivity of the airways- an immunological reaction part of a cascade which has started earlier in life. The individual had been initially sensitized through the skin.

Many studies have shown that the development of asthma in AD patients is more common, although the exact causative factors are not yet clear. In a study conducted by Pourpak et al. [12] the prevalence of asthma in patients with AD was 27,5%.

The exact pathogenesis of asthma and AD is not entirely understood. Both diseases are associated with chronic inflammation. In asthma patients cytokines and other inflammatory mediators are found in bronchial washings. Both diseases can be IgE- mediated which suggests genetic predisposition as atopy refers to the familial tendency to produce Ig-E. Imbalance in the Th1/Th2 ratio is associated with higher production of IgE in atopic patients. By producing Il-2 and Il-13, Th2 cells promote the production of IgE by B-cells in response to antigen trigger. This leads to hyperreactive response of the airways in asthma patients and in skin inflammation in AD patients. Mast cells have major role in an allergic and inflammatory disorders [13]. The many similarities between AD and asthma in terms of pathogenesis and immunological imbalance, often leads us to consider each disease in the context of the other.

A Canadian study, published in 2018 found that AD increased the risk of asthma in children at the age of 3 years only if AD was accompanied by sensitization to inhalants, foods of both at the age of 1 year [14]. Patients with severe AD, early sensitization and family history of atopy have higher risk for other atopic diseases [8]. These observations had led to the necessity of defining phenpotypes and endopypes within the AD patient population, in order to predict future development of atopic march. Classifying AD patients into phenotypes can be directly related to their risk of developing other atopic diseases in future. Based on the age of the patient: infantile, childhood, adolescent/adult and elderly AD have been defined, [15] each with its characteristic clinical features. Disease severity is often measured by SCORAD or Eczema Area Severity Index. Depending on the onset of the disease, 6 phenotypes can be distinguished: very early onset, early onset, adolescent onset, adult onset, very late onset, each group with its clinical characteristics [16]. Patients with onset of the disease late in life (over 60 years of age) usually have severe form of AD and high levels of total IgE [16]. Patients with persistent and late resolving AD most often have associated FLG-null mutations, have greatest risk of asthma, high IgE levels and atopic parent(s) [17]. Stratifying the patients according to their ethnic origin brings as well information about the mechanisms of development of the disease, its probability for evolution towards other atopic diseases and should be taken into consideration for the therapeutic decisions. For example, FLG mutations which are common in white patients with AD are not as commonly observed in South African patients [18]. FLG mutation also differ in white and Asian populations [19].

Summarized, an increased risk in developing asthma in patients with AD is related to very early onset of the AD, severe and persistent AD, early sensitization, high IgE levels, FLG null mutations, and atopic parents (or family history of atopy).

Biomarkers are measurable characteristics which have a diagnostic, prognostic or predictive value [16]. Endotypes represent the link between the pathological mechanism and the phenotype. Screening for biomarkers before the clinical signs of the disease would allow doctors to identify children with high risk of AD and atopic march [16]. Such biomarker is IgE-level. According to IgE levels for example, intrinsic and extrinsic AD are defined. Intrinsic AD is characterized by level of total IgE > 100 kU/mL [20]. This could be misleading in terms of proving allergic sensitization, because there are patients who despite the normal total IgE levels, have high specific IgE levels toward specific allergens. Bieber et al. [16] suggest that determination of the ratio of a given specific IgE level to the total Ig E would be more useful biomarker to identify the sensitization profile of a patient or the potential usefulness of a particular therapy. However, defining exactly which biomarkers to be used, or the significance of their predictive values is another challenge. Same biomarkers will not have the same predictive value in different phenotypes especially when patients are in different age groups. The maturation of the immune system and the development of tolerance over time are also obstacles for the establishment of a uniform strategy. Perhaps the solution would be to differentiate biomarkers or their predictive values according to the phenotype. Reassessment needs to be done, as diseases severity changes over time.

## Comorbidities in asthma and AD

In addition to the atopic march, atopic patients also have an increased risk of developing contact dermatitis, hand dermatitis, and irritant contact dermatitis [21–23]. Although asthma and AD are classified as type 1 allergic reaction, while contact allergic dermatitis is type 4, both represent a hyperreactivity of the immune system, as a result of an interaction with an allergen.

Apart from allergic comorbidities AD, and asthma are associated with non-allergic diseases. Non-allergic AD comorbidities consist of cutaneous and extra-cutaneous infections, neuropsychiatric conditions, obesity, cardiovascular disease, some cancers [24]. Paller et al. suggest that taking measures to reduce disease severity in childhood could have protective function against the development of these comorbidities [24]. Chronic suffering and visual manifestations of atopic diseases may significantly reduce the patient’s quality of life. The lack of long- term remissions in some patients may be the immediate cause for serious mental suffering and a predisposing factor for organic illnesses. Patients with AD are at higher risk of all- cause mortality compared to the general population [25, 26].

Th2 mediated inflammation (Il-4 and Il-13) suppresses the expression of antimicrobial peptides in AD [27]. Therefore AD patients are more susceptible to *S. aureus* and other bacterial skin infections, compared to the general population [28]. Such skin infections include- herpes simplex, and higher risk of developing eczema herpeticum [29], coxsackie virus and developing eczema coxsackium [30], more severe molluscum contagiosum infection [31], HPV-related cervical cancer [32] and chickenpox [33]. This applies not only for the skin, as inflammation in allergic asthma is also Th2 mediated, and the risk of infectious diseases is increased as well. Extracutaneous infections include respiratory, gastro-intestinal, and urinary tracts, and the risk becomes higher with the increase of the number of allergic diseases [34]. As for neuropsychiatric conditions, AD patients are more likely to have conduct problems/disorder, ADHD, emotional problems [35], anxiety, depression with AD persistence and severity increasing the risk [36]. Chronic childhood illnesses are in general associated with an increased risk for mental health disorders [37] and allergic disease is linked to autism spectrum disorder [36], Tourette’s syndrome [38] and learning delay [39]. Association with obesity has been established for AD, asthma and FA not for AR [24]. AD patients are at higher risk of coronary artery disease (CAD), again with the greatest risk for patients with severe and persistent AD [40]. Allergic disease increase the risk for autoimmune disorders such as Chron’s disease, ulcerative colitis and alopecia areata [41]. Severe forms of alopecia areata are specifically associated with AD and a greater risk when filaggrin mutations are present [42]. Although some data suggest reduced risk of malignancies in allergic disorders [43] AD is associated with increased risk of lymphoma overall [44].

The association between AD, asthma and the other atopic diseases has been well established in many reviews. Beyond the atopic march, Table 1 shows the most frequent comorbidities for asthma and AD (based on reviews of the medical literature) [24, 45].

**Table 1:** Frequent comorbidities of asthma and AD

Asthma	Atopic dermatitis
Chronic rhinitis	Infections (cutaneous and extracutaneous-respiratory)
Chronic sinusitis/rhinosinuitis	Neuropsychiatric disorders (conduct disorder, anxiety, depression)
Gastroesophageal reflux disease	Obesity
Obstructive sleep apnea/sleep-disordered breathing	Cardiovascular disease, Stroke
Psychological disturbances (depression and anxiety disorders)	Type 2 diabetes
Chronic/recurrent respiratory infections	Autoimmune disease (Chron’s disease, ulcerative colitis, alopecia areata)
COPD	Lymphoma
Hyperventilation syndrome	
Glottic (vocal cord) dysfunction	
Hormonal disturbances	
Obesity	
Smoking (tobacco addiction)	

It is obvious that asthma and AD share a lot in terms of comorbidities. Perhaps due to the immunological disturbance (Th2 cytokine expression) the resistance to infections is reduced. Obesity and psychological disturbances due to the emotional burden of chronic suffering are also common traits of both conditions. Asthma and AD should be considered systemic disorders with major organs affected respiratory tract and skin, respectively, and multiple organs and systems of secondary involvement.

## Skin barrier

Impaired skin barrier is a major factor for the development of atopic diseases as transepidermal penetration of antigens leads to sensitization. This is why in the majority of cases AD precedes asthma. Allergic asthma is the most common type of asthma and is usually defined by the presence of sensitization towards environmental allergens. It is associated with increased prevalence of AD and allergic rhino-conjunctivitis, as total IgE levels are usually higher, and there is presence of Th2 cytokines in secretions and peripheral blood of asthma patients [46]. Skin barrier is explained by the “brick and mortar” model of the stratum corneum (SC), where the corneocytes represent the bricks and the surrounding lipids are the mortar [47]. Decreased filaggrin, ceramides, antimicrobial peptides, serine protease (SP) inhibitors and impaired tight junctions (TJ) have been found to be associated with dysfunctional epidermal barrier [48].

In AD the increased pH level and elevated SP activity leads to reduced ceramide synthesis and lamellar body secretion [49]. Immunologic deviations such as increased IFN-α levels also lead to decreased synthesis of ceramides [49]. Chain lengths of ceramides, free fatty acids, and esterified fatty acids are shortened in lesional tissue, leading to epidermal lipid disorganization and abnormal skin barrier permeability [50]. Filaggrin is structural protein of the skin and plays an important role in the keratinization, moisturization and antimicrobial functions of the skin [51]. Its deficiency is associated with AD, increased sensitivity and severity of allergies and increased risk of infection [52]. Genetic abnormalities are associated with dry skin and transepidermal water loss (TEWL) [53]. However, filaggrin mutations are found in only 15–50% of the patients with AD [54]. Impaired tight junctions also contribute to the skin barrier dysfunction in AD, as they control the cellular permeability in the granule layer and represent a second physical barrier in the epidermis [55].

Application of moisturizers in the neonate period reduces the incidence of AD by 50% at 6 months [56] and by 32% at 32 weeks [57]. This external restoration of the skin barrier helps overcome the AD and prevents the development of asthma.

The concept of the impact of the environment on human health is widely discussed in recent years. The term “exposome” refers to all environmental factors to which the individual is exposed in their lifetime. The evidence of its influence on the pathogenesis and evolution of asthma and AD is rapidly growing in the medical literature. In a review published in 2018, Cecchi et al. [58] present and discuss the mechanisms by which environmental factors (such as chemical air pollutants, aeroallergens, climate changes) contribute to the development of allergic respiratory and skin diseases. Air pollutants interact with aeroallergens and increase the risk of sensitization and aggravation of allergic diseases. According to the authors environmental factors may not only lead to increased frequency but also can affect the clinical appearance of allergic diseases [58]. Industrialization is linked to abundance of epithelial barrier-damaging agents, and defective barrier is associated with allergic diseases such as asthma, AD and others.

## Microbiome

Various resident microorganisms inhabit the human skin, airways and gastrointestinal tract. A fine balance in the microbial communities exist and its distortion is called dysbiosis.

The microbiome plays an important role in both health and disease, as it takes part in the development of the immune system and the development and manifestations of allergic diseases [59]. Since the “hygiene hypothesis” [60] in the 1980’s, there is growing number of researches which link the microbiota of the skin, gastrointestinal tract and respiratory tract to allergic disease [59]. Rural environment, breast feeding, absence of early exposure to antibiotics, pets at home during childhood, maternal exposure to animals during pregnancy are associated with lower risk for allergic disease [61–65]. During postnatal period the immune system of the child is immature, therefore microbial colonization occurs without triggering an immune response [66]. Microbial colonization with commensal microorganisms is crucial for developing healthy immune tolerance. Microbial products from the normal gut, lung and skin flora have protective effect towards pathogens. Altered microbiome triggers the immune system and leads to disruption of physical barriers and penetration of pathogens and allergens.

Colonization with *S. aureus* is associated with increased IgE levels, FA and more severe AD [67, 68]. *S. aureus* produces virulence factors which act as superantigens and induce Type 2 immune response, suppress the T regulatory cells, mechanisms connected with both asthma and AD. Colonization with *S. aureus* itself is a result of an impaired skin barrier for example when FLG expression is decreased and IL-13 and IL-4 expression is increased [59]. *S. aureus* is commonly cultured from the skin of patients with AD and its persistence is associated with more severe disease (AD) flares [69]. *S. aureus* can be isolated from the nares of one third of the population [70] and its resistance to antibiotics is not uncommon [71]. *S. epidermidis* strains which express serine protease glutamyl endopeptidase (GluSE) were reported to inhibit the *S. aureus* biofilm formation [72] and also, its protease activity prevents the adhesion of *S. aureus* [73] and the b- defensin antibacterial activity [74].

Like AD, asthma is a clinically heterogenous disease with different phenotypes and etiopathogenetic mechanisms [75]. Environmental exposures to different types of microorganisms has an impact on the development of asthma. Many studies have already shown that there is a difference in the bacterial content of the gastrointestinal tract (GIT) and the respiratory system associated with asthma [76, 77]. Specific clinical phenotypes and inflammatory features of asthma, including hyper-responsiveness of the airways, obesity-associated asthma, responsiveness to corticosteroid and macrolide therapy, type 2 airway inflammation of the bronchial epithelium, are associated with different microbial populations in the lower airways [59, 78, 79].

In both asthma and AD the cross-talk between microbiota and immune system are implicated in the pathogenesis, susceptibility and phenotype of the disease. Alterations in the microbial flora lead to immune response and vice-versa. *S. aureus* is a major microbial factor associated with increased IgE levels and more severe disease flares.

## Therapy

Many new therapies targeting different parts of the immunological pathway of asthma and AD have already been developed and are available, or are in process of development [80]. Dupilumab, blocking IL-4 and Il-13 from binding to its receptors, was the first biologic approved for moderate to severe AD, and Omalizumb, anti- IgE monoclonal antibody, was the first biologic approved for the treatment of asthma in the Unated States and the European Union [81].

Th2 targeting therapy prevents the development of asthma and AD, as it is aimed at shared for the atopic march immunological components such as IL -13, IL- 4, TSLP etc. For many years the treatment of AD consisted in the use of emollients, phototherapy, caucineurin inhibitors, corticosteroids and other immunosuppressive therapies. Thanks to the numerous researches on the pathogenesis of atopic diseases new therapies have been developed, and many are under development, with potentially fewer side effects and better therapeutic response, aiming at specific pathogenetic mechanism of the disease.

Dupilumab is a monoclonal antibody for the IL-4 receptor a, which blocks the IL-4 and IL-13 pathways and increases the expression of filaggrin, loricrin and claudins [82]. Equaly efficient for asthma, Dupilumab is also used for the treatment of chronic rhinosinusitis with a nasal polyps. JAK- STAT inhibitors (baricitinib, upadacitinib, PF-04965842, ASN002, tofacitinib and others) also increase filaggrin expression and reduce inflammation, therefore restore skin barrier [83]. Targeting the JAK-STAT signaling pathway can reduce Th2 inflammation implicated in both asthma and AD. Lebrikizumab and tralokinumab, IL-4/IL-13 inhibitors, show clinical efficiency in asthma and AD [84, 85] indication for the shared inflammatory mechanisms in the atopic diseases pathogenesis.

## Conclusions

There is a strong link between asthma and AD. Systemic factors such as genetics and impaired Th2 immunity, and tissue specific factors such as local immune response, barrier dysfunction, abnormal microbiome and environmental triggers contribute to the increased risk for atopic comorbidities [8]. In general, patients with early onset, severe and persistent AD, who have family history of AD at higher risk of developing asthma. Th2 immune response, its pathways and mediators are undoubtedly the main driving mechanism in both AD and asthma. However, not every patient with AD develops asthma and not every patient with asthma is atopic. The link between those two conditions exists in the context of the atopic march where certain pathogenetic mechanisms overlap to a greater extent. Early start of a targeted therapy toward specific shared pathogenetic mechanism, could have considerable benefits for the patients at high risk, as developing allergic comorbidities is a prerequisite itself for developing other non-allergic ones. However, identification and stratification of risk groups may be the hardest part, as the definition of the significant biomarkers and their prognostic values in accordance to the phenotypic expression are still challenges that need to be overcame.

## Data Availability

the datasets generated and/or analyzed during the current review are available in the PubMed repository, https://pubmed.ncbi.nlm.nih.gov/

## Abbreviations

AD: Atopic dermatitis
FA: Food allergy
AR: Atopic rhinitis
FLG: Filaggrin
ADHD: Attraction Deficit Hyperreactivity Disorder
CAD: Coronary Artery Disease
IL: Interleukin
SC: Stratum corneum
SP: Serine Protease
GluSE: Glutamyl endopeptidase
TJ: Tight junction
IFN-α: Interferon alfa
TEWL: Transepidermal Water Loss
TSLP: Thymic Stromal Lymphopoietin
JAK inhibitor: Janus Kinase inhibitor
JAK-STAT: Janus kinases (JAKs) Signal Transducer and Activator of Transcription Proteins (STATs
